# Cervical gland area as an ultrasound marker for prediction of preterm delivery: A cohort study

**Published:** 2017-11

**Authors:** Vajiheh Marsoosi, Reihaneh Pirjani, Mohamad Asghari Jafarabadi, Mina Mashhadian, Saeedeh Ziaee, Ashraf Moini

**Affiliations:** 1 *Perinatology Division, Obstetrics and Gynecology Department, Tehran University of Medical Sciences, Tehran, Iran.*; 2 *Obstetrics and Gynecology Department, Arash Women’s Hospital, Tehran University of Medical Sciences, Tehran, Iran.*; 3 *Road Traffic Injury Research Center, Tabriz University of Medical Sciences, Tabriz, Iran.*; 4 *Department of Statistics and Epidemiology, Faculty of Health, Tabriz University of Medical Sciences, Tabriz, Iran.*; 5 *Islamic Azad University, Maybod, Iran.*; 6 *Tarbiat Modaress University of Medical Sciences, Tehran, Iran.*; 7 *Department of Endocrinology and Female Infertility, Reproductive Biomedicine Research Center, Royan Institute for Reproductive Biomedicine, ACECR, Tehran, Iran.*

**Keywords:** Labor, Preterm, Ultrasonography, Cervical length measurement, Pregnancy outcome

## Abstract

**Background:**

Preterm labor is a major cause of perinatal morbidity and mortality and it might be predicted by assessing the cervical change.

**Objective:**

To assess the association between absence of cervical gland area (CGA) and spontaneous preterm labor (SPTL).

**Materials and Methods:**

This prospective cohort study was performed on 200 singleton pregnant women with a history of SPTL, second-trimester abortion in the previous pregnancy or lower abdominal pain in current pregnancy. Each patient underwent one transvaginal ultrasound examination between 14-28 wk of gestation. Cervical length was measured and CGA was identified and their relationship with SPTL before 35 and 37 wk gestation was evaluated using STATA software version 10.

**Results:**

The mean of cervical length was 36.5 mm (SD=8.4), the shortest measurement was 9 mm, and the longest one was 61 mm. Short cervical length (≤18mm) was significantly associated with SPTL before 35 and 37 wk gestation.

Cervical gland area (the hypoechogenic or echogenic area around the cervical canal) was present in 189 (94.5%) patients. Absent of CGA had a significant relationship with SPTL before 35 and 37 wk gestation (p=0.01 and p<0.001, respectively). Cervical length was shorter in women with absent CGA in comparison with subjects with present CGA: 37±10 mm in CGA present group and 23±9 mm in CGA absent group (p<0.001).

**Conclusion:**

Our study showed that cervical gland area might be an important predictor of SPTL which should be confirmed with further researches.

## Introduction

Preterm birth (PTL) is the preceding cause of perinatal morbidity and mortality and preterm neonates are at increased risk of respiratory distress syndrome, intraventricular hemorrhage, sepsis, necrotizing enterocolitis, and long-term serious developmental problems (1-5). Thus prediction of PTL is highly important. For more than two decades ultrasound plays an important role in the obstetrics and many studies have pointed at sonographic markers to predict PTL.

Certainly, cervical change plays an important role in labor initiation, thus cervical assessment is a principal part of the prediction of PTL. Cervical length and cervical funneling are the most parameters evaluated. Dalili and coworker showed a significant relationship between cervical length at second trimester and PTL risk (6). Shortening of the cervical length reflect cervical maturation and is a valuable criterion of the PTL, however, it is not solely criterion. Indeed shortening of the cervical length is only one of the signs of cervical maturation. Despite the many studies that have been conducted on cervical length, the prevalence of preterm birth is still high, so it seems that more studies were needed on other cervical changes. The disappearance of the cervical gland area (CGA) is another alteration in cervical maturation process. Physiological cervical ripening is associated with changes in the collagen and proteoglycan composition (7). Cervical maturation is often recognized by collagen disorganization, decreased collagen concentration, and increased water content (8). Factors such as an increase in water content and biochemical changes are responsible for the disappearance of CGA in ultrasound imaging (9). This area is present in most women, pregnant and nonpregnant. However, it disappears with advancing pregnancy or earlier in PTL (9). It seems that previous studies on the CGA are still not sufficient and further studies addressing this issue are needed (10, 11).

The aim of this study was to evaluate the role of the absence of CGA in the risk assessment of PTL

## Materials and methods

This cross-sectional study was performed on pregnant women referred for Prenatal Care Center, Shariati Hospital, Tehran University of Medical Sciences, Tehran, Iran between March 2011 and September 2012. A total of 251 pregnant women were assessed. Gestational age was assumed from last menstrual period in women with regular menses and/or crown-rump-length (CRL) at the first trimester. The inclusion criteria were a singleton pregnancy with a history of spontaneous PTL or second-trimester abortion in a previous pregnancy or lower abdominal pain in the presence of uterine contractions in current pregnancy. In women who had abdominal pain, uterine contractions were evaluated and if there were at least two contractions in ten minutes they were considered as high risk. The exclusion criteria were gestational age more than 28 wk of gestation, multiple pregnancy, premature rupture of membrane, vaginal bleeding, placental abruption or placenta previa, cervical cerclage, intra uterine growth restriction (IUGR) and polyhydramnios or oligohydramnios, fetal malformation, maternal systemic disease, Mullerian anomaly, uterine contraction and nonspontaneouse (iatrogenic preterm labor. Each participant underwent one transvaginal ultrasound exam performed between 14-28 wk of gestation to assess cervical length, cervical funnelling and CGA. Although it seems that digital examination has no effect on ultrasound results, no digital examination was performed before ultrasound exam. All ultrasound examinations were performed by one perinatologist, by Acuson Sequoia 512 ultrasound machine or Medison V10 machine with a 6-10 MHZ endocavitary probe. The perinatologist was not involved in decision making and patient follow up the bladder was empty and cervical length measurement was performed in the sagittal view after visualizing the internal and external orifice in the same plane, and the presence of the CGA was evaluated simultaneously. Cervical length was measured three times and the shortest distance between external and internal orifice was recorded. The cervical funnelling was defined as the opening of internal orifice exceeding 5 mm in the width (12). 

**Figure 1:**
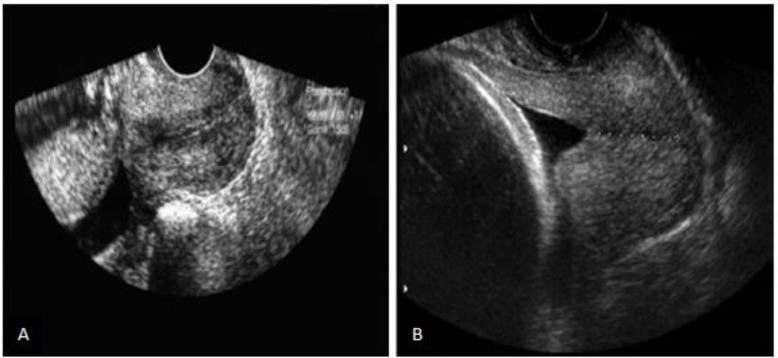
Presence of cervical gland area (A) and absence of cervical gland area (B).

The cervical gland area was defined as a hyperechoic or hypoechoic zone around cervical canal which corresponds to histological CGA (9). If the hypoechogenic or echogenic area around the cervical canal was visualized, the presence of CGA would have been established. According to this view, the pregnant women were divided into two groups based on presence or absence of CGA (Figure 1). Then pregnant women were followed up until delivery time. 

**Ethical consideration**

This study was approved by Ethics Committee of Tehran University of Medical Sciences (52D/5619).

**Statistical analysis**

All analyses were performed by STATA software version 10 (StataCorp, College Station, Texas). Data were presented as frequency (percentage), median, or mean (±SD) based on the nature of the variables. Independent samples *t*-test, Mann Whitney test, and Fisher exact test were applied to analyze differences between absent and present CGA groups. 

Univariate and Multivariate analyses logistic regressions were performed for investigating the relationship between cervical markers (cervical length, cervical funnelling, CGA) and SPTL before 35 and 37 wk gestation. Due to low number of women with PTL and instability of results in the ordinary logistic regression, we shift to use exact logistic regression. SPTL before 35 and 37 wk gestation were considered as binary dependent variables. Multivariate analyses were used to adjust for confounding variables including age, gravid, PTL history, abortion history and pregnancy age at ultrasound examination time. In addition, the cut-off value for cervical length was determined by receiver operating characteristic (ROC) curve. The area under the curve and their 95% CI was reported as the prediction adequacy of cervical length for SPTL. P-value<0.05 was considered as significant.

## Results

A total of 251 pregnant women were entered into the study. Of whom 29 (11.5%) women were removed because of having some exclusion criteria and 22 (8.7%) women missed to follow up, therefore 200 (79.8%) pregnant women remained for final analysis including 169 (84.5%) women with lower abdominal pain in current pregnancy, 10 (5%) women with previous PTL or second trimester abortion and 21 (10.5%) women with both of them. The mean age of patients was 27±6 yr. The number of gravid varied between one and three. Mean gestational age at ultrasound examination time was 20.25±3.52 wk. A comparison of some background characteristics including age, gravid, PTL history, abortion history, abdominal pain, cervical length, and gestational age at ultrasound exam between the CGA absent and present groups are shown in table I. 

**Figure 2:**
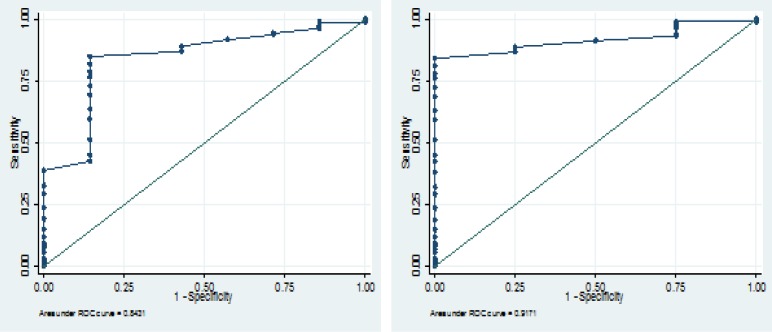
ROC curves for determining the cut-off values of cervical length for SPTL before 35 wk (left) and 37 wk (right

Among the total 200 women, 7 (3.5%) patients presented SPTL before 37 wk gestation, and four (2%) patients presented SPTL before 35 wk of gestation. The mean of cervical length was 36.5 (SD=8.4) mm, the shortest cervical length was 9 mm and the longest was 61 mm. We found 18 mm as a cut-off value by ROC curve for selected sensitivity and specificity for SPTL before 35 and 37 wk gestation. Area under curve (and it’s 95% CI) were 0.843 (0.703-0.983) and 0.917 (0.859-0.975) for SPTL before 35 and 37 wk gestation respectively (Figure 2). 

In three (1.5%) of patients, cervical length was less than 18 mm. Short cervical lengths (≤18mm) was significantly associated with SPTL before 35 and 37 wk gestation (Tables II, III). The cervical funnelling was seen in 18 (9%) of women. This morphologic parameter had a statistically significant relationship with SPTL before 35 wk but did not have such relationship with SPTD before 37 wk of gestation (Tables II, III). 

CGA was present in 189 (94.5%) patients. In 5.5% (11 women) of total women who were studied, CGA was absent including four women with SPTL before 35 wk of gestation, and six women with SPTL before 37 wk of gestation. Absent of CGA had a significant relationship with SPTL before 37 and 35 wk gestation (Table II, III). Sensitivity, specificity and their corresponding positive predictive value (PPV) and negative predictive value (NPV) were 25.0% and 99% respectively (PPV=33.3% and NPV=98.5%) for before 35 wk gestation. 

The sensitivity and specificity were 14.3% and 99.0% respectively (PPV=33.3% and NPV=97.0%) for 37 wk gestation. Our result showed that cervical length was shorter in women with absent CGA in comparison with subjects with present CGA (p<0.001) (Table I).

**Table I.** Demographic characteristics in two study groups (CGA present and CGA absent

**Variables**	**CGA present group**	**CGA absent group**	**p-value**
Age (yr) #	27 (24-31.5)	29 (27-38)	0.06
Gravid #	1 (1-2)	2 (1-3)	0.17
Preterm labor history*	15 (7.9)	2 (18.2)	0.23
Abortion history*	4 (2.1)	0 (0.0)	0.79
Abdominal pain*	180 (95.2)	10 (90.9)	0.44
Cervical length (mm)**	37.3 (7.6)	21.7 (6.5)	<0.001
Gestational age at ultrasound exam (wk)**	20.2 (3.5)	21.0 (3.9)	0.46

CGA: Cervical gland area.

# **:** Present the median (Q1-Q3) as summary statistics and the results of mann-whitney U–test for comparison

* **:** Present the frequency (%) as summary statistics and the results of fisher’s exact test for comparison

**: Present the mean (SD) as summary statistics and the results of independent samples t-test for comparison

**Table II:** Results of univariate and multivariate exact logistic regression of three sonographic markers for spontaneous preterm labor before 37 wk gestation

**Variable**	**Unadjusted**		**Adjusted**	
	**OR (95%CI)**	**p-value**	**OR (95%CI)**	**p-value**
Cervical length	0.87 (0.79-0.95)	0.002	0.91 (0.82–0.99)	0.035
Funnelling	4.36 (0.55-24.11)	0.140	3.41 (.25-91.42)	0.351
Cervical gland area	195.87 (24.12-5215.00)	<0.001	278.54 (32.73-inf)	<0.001

OR: Odds ratio

CI: Confidence Interval Mid-p-value computed for the probabilities and CIs based on exact logistic regression analyses

Inf: Could not be computed due to low number of events in CGA present category

**Table III:** Results of univariate and multivariate exact logistic regression of three sonographic markers for spontaneous preterm labor before 35 wk gestation

**Variable**	**Unadjusted**		**Adjusted**	
	**OR (95%CI)**	**p-value**	**OR (95%CI)**	**p-value**
Cervical length	0.85 (0.75-0.95)	0.004	0.89 (0.78-0.99)	0.047
Funneling	10.95 (1.08-111.10)	0.044	1.00 (0.05–inf)	0.005
Cervical gland area	121.47 (19.35-inf)	<0.001	14.48 (2.20–inf)	0.011

OR: Odds ratio

CI: Confidence Interval Mid-p-value computed for the probabilities and CIs based on exact logistic regression analyses.

Inf: Could not be computed due to low number of events in CGA present category

## Discussion

The results showed that the odds of PTL before 35 and 37 wk of gestation was significantly increased in pregnant women with absent CGA at 14-28 wk gestation in both univariate and multivariate exact logistic regressions. Furthermore, cervical length was significantly shorter in women with absent CGA comparing with present CGA. Our results are similar to several other studies that showed the importance of CGA for prediction of PTL (9, 13-16). Fukami and coworker evaluated the relationship between cervical length and CGA at 16-19 wk gestation with early PTL and reported 99.8% specificity and 75% sensitivity for early PTL using CGA (13). Asakura *et al* concluded that short cervical length with absent CGA displays an independent predictor for PTL in patients with threatened PTL and noticed that detection rate of CGA were more than 90% between 16-31 wk gestation and then decreased to 16% at 40 wk gestation (14). Pires evaluated the association of SPTL with cervical length, the absence of CGA and cervical funnelling in general population. Their results represent that the absence of CGA can be an important ultrasound marker for SPTL (15).

Although all of the previous studies considered the absence of CGA as a novel and impressive marker for PTL prediction, but incidence of absent CGA is very different among these studies, for example, 0.36% between 16-19 wk gestation reported by Yoshimatsu *et al* (16) and 2.7% between 21-24 wk gestation reported by Pires and coworker (15) and 5.5% between 14-28 wk gestation in our study. The different incidence of absent CGA among these studies may be due to the difference in study design and choice of subjects. In 2013, 85 women with threatened PTL symptoms were examined by Kahyaoglu *et al* (10). They demonstrated the presence of echogenic (not echolucent) CGA on transvaginal ultrasonography is associated with PTL among patients with a short cervix that is in contrast to our results. However, their sample size was low. 

There is no consensus about other cervical markers. For example, despite the vast use of 

cervical length measurement, there is no definite cut-off for short cervix. This range is from less than 15 mm to 35 mm (12, 17-21) so that we found a cut-off value of 18 mm for prediction of PTL. Similarly, cervical funnelling is often due to uterine contraction and cannot be considered a valid predictor of PTL (16). Against funnelling, CGA straightly reflects cervical maturation and is usually less subjective (16). Yamaguchi *et al* evaluated CGA and cervical length at 37-38 gestational age and showed that CGA in combination with cervical length is a useful predictor for the onset of labor within a week in pregnant women at term (11). Recently, the researchers show interest to present new techniques that could be used to evaluate cervical changes. Parra-Saavedra *et al* (22) newly defined CCI (cervical consistency index), a new TVS (transvaginal sonography) technique, and assess its potential value in the prediction of SPTL. Cervical length, gestational age, maternal weight and BMI (body mass index), parity and age must be considered to determine this index and 5 steps must be performed. In addition, reference ranges for CCI is not established yet. McFarlin *et al* introduced a new technique for evaluation of cervix (8). They detected dynamic changes in cervical structure by estimating changes in ultrasonic attenuation. Although this is an interesting method, however, requires a special device and software. Unlike these recent methods assessment of CGA requires no special technology and is easy to do (8, 22). Various studies have emphasized the increasing importance of CGA as a PTL predictor (9, 14-16). Nevertheless, early prediction of SPTL remains a major concern for researchers. Further studies are needed to confirm the significance of absent CGA in the prediction of PTL. 

In our study only 3.5% of women delivered before 37 weeks of gestation that it seems very low. In our previous study we obtained 7.7% PTL in our general population (23). We believe that the rate of PTL in this study is very low. According to inclusion criteria, a large number of our population had lower abdominal pain. This sign is common in pregnancy and most of the times is not related to cervical shortening or dilatation, thus our population was not high risk for PTL. Furthermore, bed rest, administration of progesterone and cerclage were used if indicated, that may explain so low preterm birth rate in this study. One of the limitations of our study is the low numbers of subjects which makes the CI’s very wide and un-interpretable and could be corrected with a higher number of subjects in the larger studies or multicenter studies. As another limitation of this study, was small values sensitivities for determining the cut-off value for cervical length. Further limitation is that only 15.5% of participants were really high risk for PTL and others had lower abdominal pain that cannot be considered high risk for preterm labor. 

We suggest more detailed studies on high-risk population groups and also on general population with larger sample size to clarify the accuracy of absent CGA as a valid predictor of PTL.

## Conclusion

In conclusion, our results indicate a significant association between absent CGA at 14-28 wk gestation and SPTL before 35 and 37 wk gestation. Absent CGA reflect the cervical maturation and might be a strong predictor of PTL which must be confirmed with further studies.
